# Developing a reliable predictive model for the biodegradability index in industrial complex effluent

**DOI:** 10.1038/s41598-025-15866-0

**Published:** 2025-08-17

**Authors:** Sadegh Partani, Amin Arzhangi, Hamidreza Azari, Hamidreza Moheghi

**Affiliations:** 1 Faculty of Engineering, Civil Engineering Department, University of Bojnord, Northern Khorasan, Iran; 2 Environmental Engineering Department, Centrale Méditerranée, Marseille, Provence- Alpes-Côte d’Azur, France; 3 Faculty of Engineering, Industrial Engineering Department, University of Bojnord, Northern Khorasan, Iran

**Keywords:** Industrial wastewater, Industrial park, Biodegradability index, Breusch-Pagan test, Multivariate linear regression, Backward elimination regression, COD/BOD_5_ ratio, Environmental prediction, Environmental chemistry, Environmental impact, Civil engineering, Biochemistry, Environmental sciences, Natural hazards, Chemistry, Engineering

## Abstract

The interaction between chemical oxygen demand (COD) and biological oxygen demand (BOD5) in wastewater from Tehran’s Paytakht and Nasirabad Industrial Parks is investigated in this work. Monitoring platforms of industrial parks were the base frame of monthly collection data for laboratory measurements (for BOD5 and COD) and in-situ measurements (for DO, EC and Temperature-T°C) with a frequency of 4-hour samples/day. Backward elimination regression analysis was employed as an integrated procedure to find out effective model removing ineffective independent variables. Multivariate Regression analysis showed a relatively strong linear relationship between COD and BOD, with independent variables with R²=0.64 and R²=0.59, respectively. A prediction model for BOD based on COD was found by analyzing important effluent quality variables using simple linear regression and a strong linear association (BOD = 0.433COD + 222) with R² = 0.94, MSE = 38,829, RMSE = 197.05 was obtained. In all of these regression analyses, model accuracy was assessed by conducting statistical tests on the residuals. To verify and improve the reliability and practicability of model, it is applied of industrial parks’ wastewater records of countries around the world such as Egypt, France, India, Pakistan and Malaysia. The extracted model applied on some of the mentioned countries’ records and the results of BOD prediction was matched by observations in 95% of reliability domain. Variation of BOD-COD ratio was least affected by pH and temperature; the results underline the requirement of localized validation resulting from industry-specific differences and promote cost-effective, quick wastewater evaluation, hence lowering reliance on laboratory-based BOD_5_ testing. It defiantly provides the opportunity of analytical and applied researches in south countries toward sustainable industrial wastewater management.

**Supplementary Information:**

The online version contains supplementary material available at 10.1038/s41598-025-15866-0.

## Introduction

Wastewater varies widely in its composition, and these variations play a critical role in determining appropriate identification and treatment strategies^1^. Each type of wastewater, such asdomestic^2^ industrial^3^ and agricultural^4^ introduces unique chemical, biological, and physical characteristics. For instance, industrial wastewater sometimes includes significant amounts of harmful chemicals, heavy metals^5^ or organic pollutants, in contrast to domestic wastewater, which usually consists of organic matter and nutrients^6^. In assessing the pollution load of wastewater, chemical oxygen demand (COD) and biological oxygen demand (BOD_5_) serve as key indicators of its contamination level and overall environmental impact. BOD_5_, the oxygen required by microorganisms to break down organic compounds in the water and wastewater, is a main sign of organic pollution in aerobic environments^7^. It can also be used as one of the biodegradability indices (BI)^8^. BI is often calculated using various ratios, such as BOD₅/TOC^9^ COD/TOC^10^ BOD₅/Total Nitrogen (TN)^11^ BOD₅/Total Phosphorus (TP)^12^ and most commonly, BOD₅/COD^13^. On the other hand, COD measures the total amount of oxygen required for the chemical oxidation of both organic and inorganic substances in water, particularly in industrial wastewater^14^. It can also be considered one of the chemical degradability indices^8^. Taken together, These parameters provide a comprehensive view of the potential negative impact of wastewater^15^ enabling environmental engineers and environmental scientists to monitor compliance with regulations, develop effective treatment strategies, and assess water quality. High BOD_5_ and COD levels often cause low dissolved oxygen (DO)^16^ in water, which can harm aquatic life and understanding and managing these factors is essential for the sustainable management of water resources. Optimizing municipal wastewater treatment helps to demonstrate their efficiency by removing COD and BOD_5_ using granular activated carbon (GAC)^17^. The research obtained removal rates of up to 91% for COD and 93% for BOD_5_, underscoring the importance of surface area and porosity in adsorption by using GAC with different dosages (0.15, 0.20, and 0.25 g/L) and contact times. Additionally, artificial neural network (ANN) modeling supported by genetic algorithms effectively predicted treatment outcomes and optimized operational parameters, providing a cost-effective and sustainable approach with remarkable accuracy and significant environmental benefits^17^.

Another study carried out in South Sulawesi, Indonesia, reported average BOD_5_ and COD concentrations of 7.99 mg/L and 18.47 mg/L near to laying chicken farms outside of authorized limits of 2 and 10 mg/L. This emphasizes both the need for improved environmental management to address water pollution associated with poultry farming^7^. The Bat algorithm-enhanced Extreme Learning Machine (ELM-Bat) hybrid model analyzes BOD₅ in municipal wastewater treatment plants (WWTPs), thereby improving the 2023 Extreme Learning Machine approach^18^. The research^12^ emphasizes the requirement of wastewater quality modeling for management and expansion of WWTP, Including COD, pH, total suspended solids (TSS), T°^C^, DO, electricity conductivity (EC), wastewater flow, and historical effluent quality variables (EQVs). The ELM-Bat model outperforms Random Forest Regression, Gaussian Process Regression, and Multilayer Perceptron Neural Networks in predicting BOD₅ in municipal wastewater treatment, particularly when all input variables are included. The ELM-Bat significantly improves prediction dependability, environmental preservation and wastewater treatment. Twelve regression models for COD prediction in petrochemical wastewater were investigated^19^. Among them, standard linear regression achieved a correlation coefficient of 0.835 and a mean squared error of 0.041. These results highlight the advantages of advanced statistical modeling over conventional laboratory techniques, enabling more efficient and cost-effective wastewater quality control. This research also contributes to the field of environmental engineering by promoting innovative approaches to wastewater management.

Although BOD_5_ is considered an indicator for evaluating the performance of various treatment units in^20,23,24^ previous studies on BI for industries were focused on individual service industries such as Tourism^22^medical centers^21^ laundry^25^ pulp mill^26,27^ textile^28^tannery^29^ instead of industrial complexes of manufactures dominantly.

Usually, wastewater treatment is effective even if some BOD_5_ levels surpass allowed limits. Emphasizing TOC, anionic surfactants, and continuous monitoring to evaluate organic contamination in released waters, the research.

The effects of hydrogen peroxide (H₂O₂) on BOD_5_ readings in wastewater during ionizing radiation treatment are investigated^30^. Particularly at concentrations above 1 mg/dm¹, their investigation reveals that H₂O₂ distorts BOD_5_ readings due to its toxicity to microorganisms, hence causing extended adaption times and lower BOD_5_ results. The authors underline the need to consider H₂O₂ interference in BOD_5_ calculations to prevent underestimating the biodegradability of treated wastewater, thereby improving the dependability of environmental monitoring findings. Rapid, in-situ measurement of COD provides a major benefit over BOD_5_ for instantaneous water quality monitoring; hence, researchers and professionals have aimed to build a consistent relationship to forecast BOD_5_ depending on other factors. Municipal wastewater treatment plant data largely generates the existing BOD_5_-COD correlations. By means of a more accurate and precise prediction model for BOD_5_ based on COD, effluent quality monitoring would be much improved, so saving time and money and raising monitoring frequency.

Over recent years, researchers^30^ have tried to find the relation between BOD_5_ and COD to reach techniques to faster, more sensitive, and more environmentally friendly measurements^31^ in labs. Some researchers tried to find out TOC using BOD_5_ and COD^32^ or develop instrumental measurement methods for BOD_5_ prediction^33–36^. BOD_5_-COD ratio is introduced as a river pollution indicator^37–39^. All research have focused on analytical and instrumental methods to reach a faster and more precise amount of BOD_5_ in urban wastewater^40–42^ and rivers^43^ dominantly. This research is going to introduce a statistical and argumenta approach to prediction of BOD_5_ based on COD. Since COD measurement is very quick, then if the prediction of BOD_5_ be reliable, pollution level and indices can be illustrated in industrial wastewater. Recent algorithms in prediction of BOD_5_ have been published based on multiple input variables^41,44^ using machine learning and data mining methods^14,18,45^. COD prediction in industrial wastewater has been predicted in the Grey Prediction Model^46^ as an empirical analysis. ANN as a black box model was also employed to predict BOD_5_ and COD for a specific refinery^47^ without applying it to other similar refineries.

This work provides several equations for estimating BOD_5_ and COD ratio based on EQVs and explores the interaction between BOD_5_ and COD in industrial wastewater. This study underlines not only the consequences of EQVs but also the part several sectors play in the interaction between BOD_5_ and COD in industrial wastewater discharges. The possibility of general reliable BOD_5_-COD ratio provides establishing the vast variety of researches in industrial wastewater monitoring and management. Prediction of BOD_5_ typically targets varied objectives but has been studies mostly in urban or synthetic wastewater. However, the industrial wastewater has to be specifically studied because of its particular components. This work will fill the gap of industrial wastewater. In this research, through application of results on similar industrial parks’ wastewater around the world, reliability can be increased. Choosing minimal predictor variables can make possible flexible, low-cost monitoring. BOD_5_—time-consuming to analyze—then might be measures less frequently with accurate predictions. The relationships between BOD_5_ and COD could be a technical predictor for considering COD and BOD_5_ as a coupled quality variable which is discussed in the research argumenta in scientific exclusive methods with a validation on different case studies worldwide.

## Materials and methods

### Case study

**Fig. 1:**
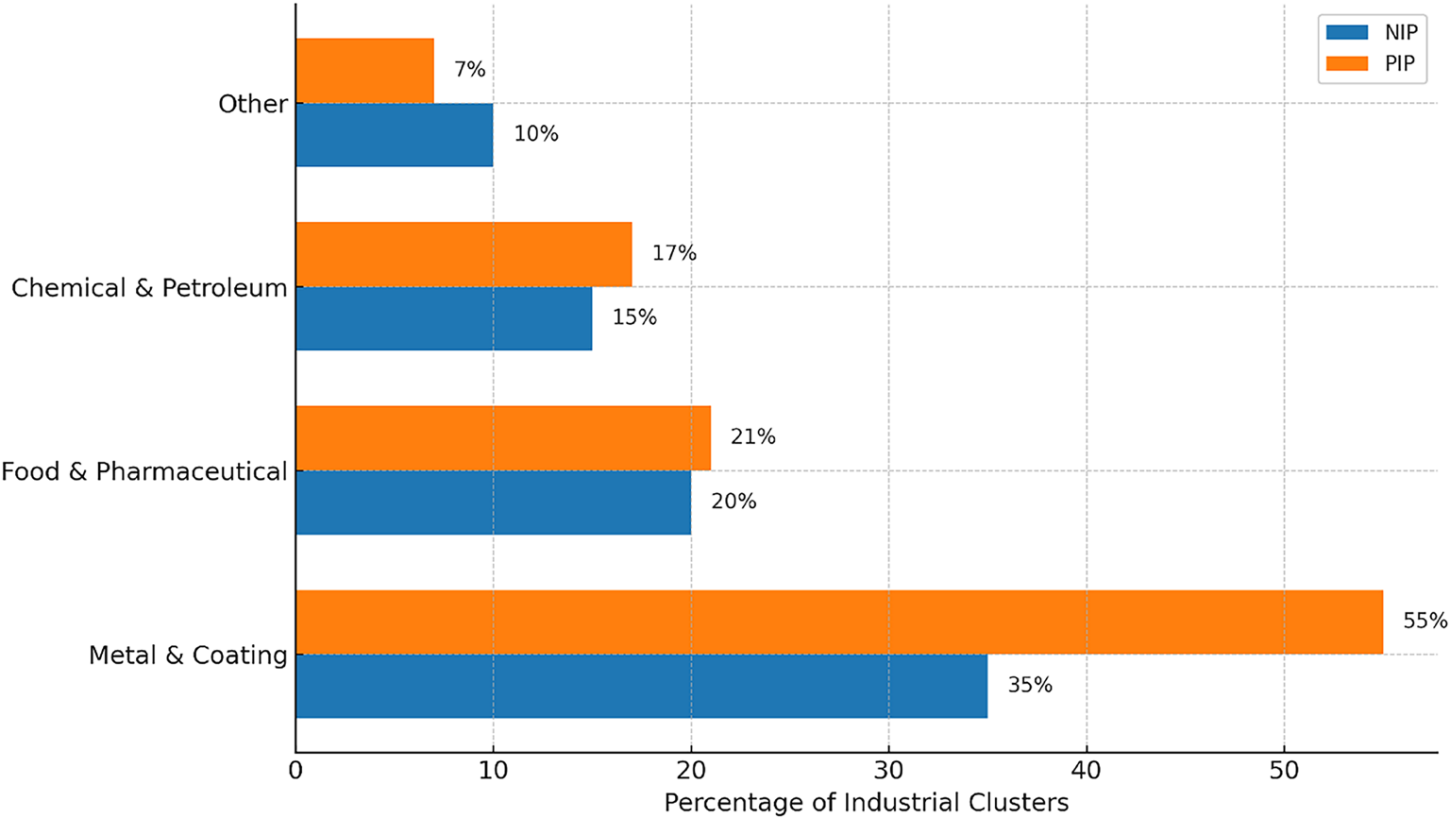
Distribution of each industrial cluster in NIP & PIP.

Located in Tehran, Iran, both Paytakht Industrial Park (PIP) and Nasirabad Industrial Park (NIP), this study concentrated on the wastewater from both of the two industrial parks^48^. Tehran, the capital, deals with major issues like overpopulation, air pollution^49^ and water contamination brought on by the great concentration of enterprises and factories inside its borders^50^. Among the most important and comprehensive industrial centers in Tehran, the selected industrial parks, NIP and PIP, were chosen due to their prominence and diversity. These parks encompass a wide range of industrial clusters, including metal and surface coating, food and pharmaceutical, as well as chemical and petroleum sectors. Their industrial composition reflects a representative cross-section of typical industrial activities found in many similar parks both within the region and internationally. Therefore, they were considered suitable for developing and testing the proposed model in this study. The industrial activities at these sites discharge a range of potentially harmful pollutants into the environment, including pathogenic microorganisms, toxic chemicals, and heavy metals. With 693 industrial units and 370 hectares, PIP consists mostly of food and pharmaceuticals (21%) followed by metal and coating (55%). Conversely, NIP occupies 243 hectares and comprises 366 industrial units comprising metal and coating (35%) and food and medicine (20%) (Fig. 1). Its main clusters are as follows: whereas NIP has a single treatment plant conveying 200 m³/s of water, PIP has two treatment plants with a total capacity of 460 m³/s. Two important measurements of wastewater quality are BOD_5_ and COD and these criteria reveal the concentration of chemical^51^ and organic^18^ components found in the wastewater. Particularly crucial is the ratio of BOD_5_ to COD since, for all kinds of wastewaters, including industrial wastewater, it stays very consistent within a given range^52^. Effluent discharge^53^ and industrial activity^54^ can affect this ratio. Finding the BOD_5_/COD ratio helps one to evaluate wastewater quality^55^ and project one parameter depending on the other.

### Field sampling and measurement

Sample size was the first step of the research that has been carried out through following equations Eq. 1. The n as sample size found out 31 for primary experimental design.

1 $$\:n=\frac{{\left({Z\:}_{\frac{\alpha\:}{2}}+{Z\:}_{\beta\:}\right)}^{2}}{{f}^{2}}$$

To account for the larger size and higher industrial discharge in PIP compared to NIP, the sampling frequency in PIP was intentionally designed to be twice that of NIP—every 5 days versus every 10 days, respectively. This frequency adjustment was incorporated within the broader framework of a temporal block design, ensuring both statistical balance and operational relevance.

To enhance the quality of sampling and ensure coverage of operational variability, a temporal block random sampling design was implemented. Each sampling day was divided into six 4-hour shifts. Within each shift, the sampling time was randomly selected from eight 30-minute sub-intervals. This design ensured the randomness of sampling while capturing effluent characteristics across all active industrial operations throughout the day. Such stratified-random timing eliminated potential confounding from temporal fluctuations and made the procedure reproducible and statistically sound.

Data collection was conducted through field sampling using a monitoring platform. The sampling frequency was every 5 days for PIP and every 10 days for NIP. Samples for laboratory analysis were collected manually, following the guidelines of EPA (2023)^56^ for wastewater sampling. In-situ measurements, such as T°^C^, DO, and electrical conductivity (EC), were performed using multiparameter instruments: Hanna HI9142 (S/N: 0750005101) and Hanna HI9811-51 (S/N: 01430006131).

Regular sampling was carried out over the course of one year. At the PIP site, 71 sampling events were conducted at 5-day intervals, while 33 events were conducted at the NIP site at 10-day intervals. On each sampling day, wastewater samples were collected every four hours, resulting in six samples per day. These samples were used to calculate the daily average concentrations of key parameters.

The primary measured variables included pH, BOD_5_, COD, T, and Total TSS (Tables S7 and S8). Aggregating multiple samples into daily averages helped reduce the data volume for analysis, while this approach may slightly decrease precision^57^ it captures short-term fluctuations throughout the day and enhances representativeness^58^.

BOD_5_ analyses were performed using the OxiTop^®^ Control BOD_5_ Respirometer System^59^ in accordance with Standard Method 5210 B (SM 5210 B for BOD_5_ and SM 5220 B for COD). Changes in air pressure within 500 cm³ brown bottles were monitored at a controlled T of (20 ± 3) °C. These pressure variations reflect oxygen consumption by microorganisms and carbon dioxide production during their metabolism.

pH was measured according to the LAB-QP-36 method, and TSS was determined using LAB-QP-25 ^19^. All laboratory procedures adhered to established standard methods to ensure consistent and accurate results. All field and laboratory equipment were calibrated during each sampling session. To verify repeatability and accuracy, random checks were conducted using a DR5000 spectrophotometer (HACH, USA).

### Quality assurance and quality control (QA/QC)

A QA/QC protocol^60^ was applied to ensure data accuracy and consistency, including duplicate sampling and use of a single trained sampler. All field and lab equipment were calibrated during each session. Random repeatability checks were performed using a DR5000 spectrophotometer (HACH, USA).

### Statistical analysis

Initially, Box Plot outlier data are discovered^61^ and removed to ensure the dataset is clean and shows the underlying trends. This phase lessens the impact of strong values that could distort the results of analysis. Once the outliers have been eliminated, the ANOVA single-factor test is next applied for a general data analysis^46^. This test evaluates if the means of several groups have statistically significant deviations, therefore providing information on the variability and potential factors influencing the data^62^. Together, preprocessing and statistical testing guarantee a powerful and reliable result interpretation. First utilized to investigate relationships between variables and develop an experimental design was Hierarchical Median Clustering Analysis^63^. This analysis revealed that various variables, including BOD_5_ and COD, show high similarity, implying common underlying elements or patterns. To thus validate these results, a pairwise correlation plot^64^ was developed to offer a complete picture of the pairwise relationships among all measured variables of PIP and NIP (TIP). The consistent findings of the correlation study matched the clustering analysis, therefore supporting the observed correlations between specific variables. Extensive multivariate analysis methods were applied, building on these realizations. Every trend and pattern found throughout the datasets was examined using a matrix plot, which offers a full picture of the data structure. The correlations between the variables were obtained using a stepwise backward elimination multiple linear regression (BEMLR) model^65,66^ on combined data of TIP. Low p-values on the study and high R² values showed a favorable correlation between BOD_5_, COD, and other EQVs after the BEMLR process.

The biodegradability model used to predict BOD_5_ based on COD was derived through simple linear regression, validated by a high coefficient of determination (R² = 0.94). This model was selected due to its statistical robustness, interpretability, and minimal data requirements, making it suitable for rapid in-field assessments where COD is readily measurable but BOD_5_ testing is time-consuming.

### Experimental design

The experimental design followed a structured plan to ensure consistent, repeatable, and valid data acquisition from industrial wastewater sources. Sampling spanned a full calendar year (2023), covering seasonal variations in both industrial parks (PIP and NIP). Samples were collected at defined intervals—every 5 days for PIP and every 10 days for NIP—with six subsamples taken across a 24-hour period to average daily values and capture diurnal variations.

To ensure the accuracy and dependability of the data, a comprehensive quality assurance (QA)/quality control (QC) inquiry (Philipp et al., 2022) was carried out. The QA/QC criteria consisted of single sampling, duplicate sampling, and choosing a certain field research group using one. Moreover, random duplicate tests were done to verify consistency, and one device was used in laboratory trials.

The study employed a stratified time-based sampling approach with replication to maintain statistical rigor. All parameters—BOD_5_, COD, pH, T, and TSS—were measured in accordance with standard procedures (SM 5210 B for BOD_5_ and SM 5220 B for COD), using calibrated laboratory instruments with QA/QC protocols.

**Fig. 2:**
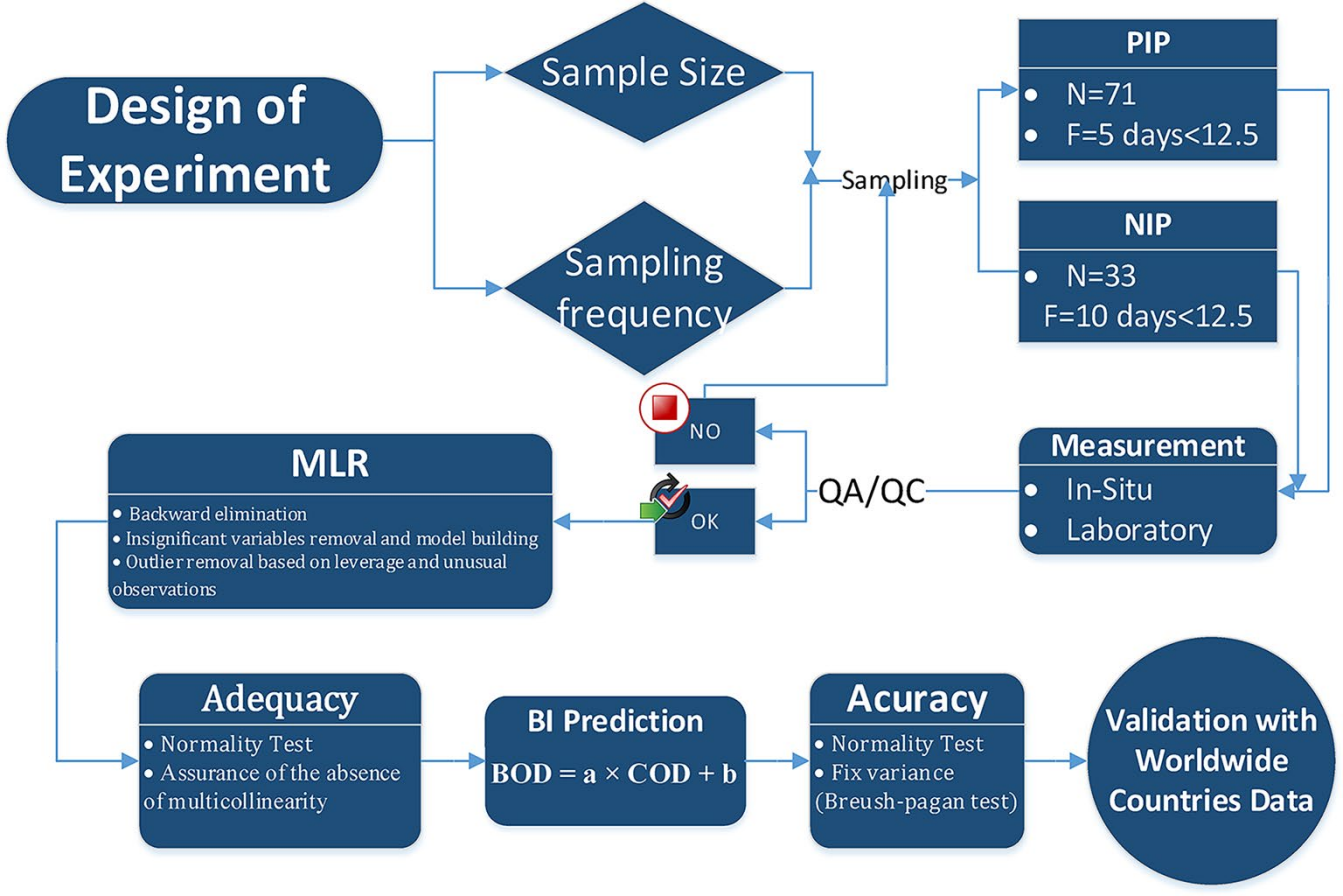
Schematic flow diagram of the experimental procedure for wastewater sampling, analysis, and modeling.

The dataset was cleaned of outliers using boxplot analysis, followed by ANOVA for primary screening. A combination of multivariate methods—hierarchical clustering, matrix plots, Pearson correlation, and backward elimination regression—was used to explore relationships and develop prediction models. Using a box plot analysis^67^ outliers were identified; around 7% of the data judged to be outliers were eliminated (Fig. 2).

### Application of equation on other industrial wastewater in worldwide countries

These results emphasized the model’s dependability and strength in identifying the hidden trends of the data. Using records of other countries, a prediction model generated from BEMLT was applied to the BOD_5_ and COD data to evaluate the dependability of these equations as the new examples. Using statistical analysis of residues and the best prediction interval for fresh examples, one may investigate the validity of the model and find This work’s primary objective was to analyze the BOD_5_-COD ratio; hence, only the equations with BOD_5_ and COD by themselves were selected for further study. The equations showed amazing efficiency and pragmatic significance in predicting industrial wastewater properties throughout many geographical areas. These findings indicate the potential of the produced equations to be a valuable tool in global wastewater management and analysis.

## Result and discussion

### Wastewater quality variables and primary screening

Table 1 compiles the average values, variances, and coefficients of variation for significant EQVs (BOD_5_, COD, TSS, T, DO, and pH) across TIP combined with their merged dataset following outlier removal. Variations in the combined dataset mirror those in BOD_5_, COD, TSS, T°C, DO, and pH. The columns include the average values, the count of observations (Count = 104 for all parameters), and the coefficients of variance for each parameter.

**Table 1:** Summary statistics of key wastewater quality parameters, including average values and coefficients of variation (CV%), based on daily composite samples collected over the monitoring period.

Groups	Count	Average	coefficients of variation %
BOD_5_ (mg/l)	104	1187.462	606132.9
COD (mg/l)	104	2252.423	3,122,134
TSS (mg/l)	104	295.4423	35034.77
T°C	104	12.37788	57.58684
DO (mg/l)	104	6.670192	66.48425
pH	104	6.635865	0.852024

**Fig. 3:**
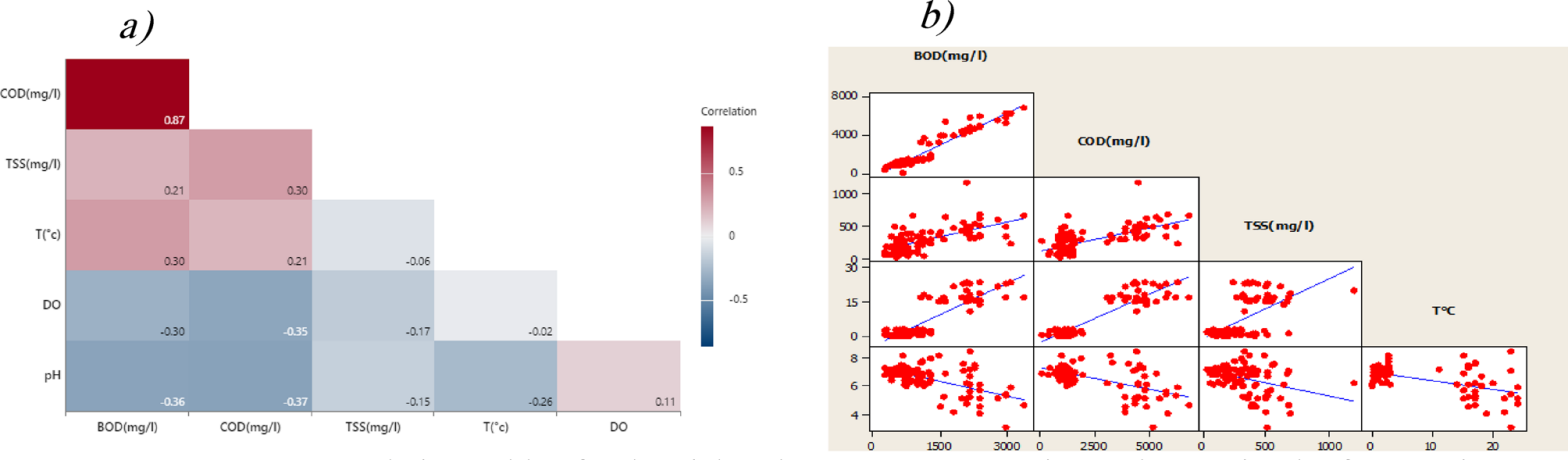
**a**) Correlation table of industrial park wastewater data in TIP **b**) Matrix plot for data in TIP.

A strong association between BOD_5_ and COD was revealed by the Pearson correlation coefficient (Fig. 3a), which was higher than 0.80 between these two parameters^68^. Conversely, the Pearson correlation value between BOD_5_ and COD with T is less than 0.35, meaning that differences in BOD_5_ and COD levels are not particularly influenced by T variations between multiple seasons or between day and night. Moreover, low is the correlation between pH and both BOD_5_ and COD, implying that pH has little influence on these values^69^.

To quantify these relationships, a backward elimination multiple linear regression (BEMLR) analysis was conducted using the measured EQVs. Based on this analysis (Table S1), predictive equations for both BOD_5_ and COD were developed (Table 2). These models demonstrated statistically significant performance, with p-values less than 0.001 ^73^.The COD prediction models achieved R² values approximately 0.64, indicating a good fit, while the BOD_5_ models showed R² values around 0.59, which is considered moderate but acceptable given the complexity of industrial wastewater. Equations 1 and 3 included the full set of variables, whereas Eqs. 2 and 4 excluded T, resulting in simpler models with three independent variables. Despite the modest R² values, all regression coefficients were statistically significant, validating the model structure and its potential application in practical monitoring scenarios.

**Fig. 4:**
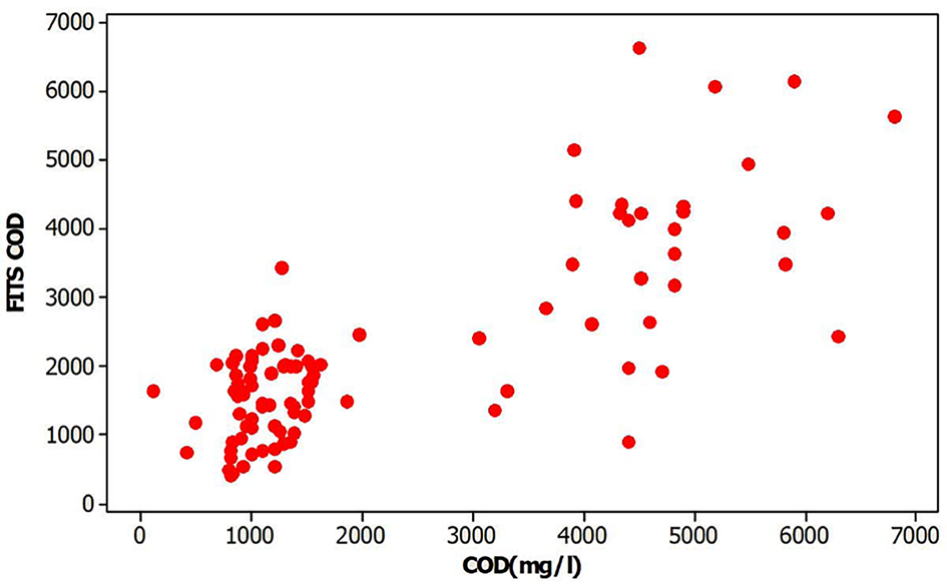
COD observed and fitted values.

T exhibited minimal influence and was therefore excluded from the final model, while the remaining three independent variables were retained. Accordingly, the regression analysis was conducted using these three variables (Table S5). All coefficients were found to be statistically significant. The analysis of variance for the model confirmed its overall performance. The resulting R² value was 0.604, which is considered acceptable, and the p-value was less than 0.0001, indicating the statistical adequacy of the multiple regression model. The relationship between predicted and observed COD values demonstrated strong agreement, as reflected by the clustering of data points along the 1:1 line in the corresponding scatter plot (Fig. 4).

### Derived MLR equations

Between BOD_5_ and COD and other EQVs, a BEMLR analysis was performed following evaluation of the EQVs and measurement accuracy. Based on the EQVs (Table 2), this study produced prediction equations for BOD_5_ and COD, which showed reasonable performance with p-values less than 0.001^73^. While the R² values for BOD_5_ were roughly 0.6, indicating a moderate-to-strong match, the R² values for equations developed for COD were above 0.6, indicating a strong fit. Equations 2 and 4 featured the most parameters; Eqs. 3 and 5 have their variable T eliminated, so leaving three independent variables in the model; these equations included different numbers of EQVs. Using these remaining factors, the regression produced an R² value of 0.64 and 0.59—considered reasonable. Furthermore, the p-value is less than 0.0001, meaning the model fits the data statistically significantly. Further verifying the correctness of the model, all coefficients in the regression output have meaning (Table 2, Table S1, and S4).

**Table 2:** Extracted primary prediction equations from BOD_5_ and COD from BEMLR between EQVs for data in total records.

Equations Number	Primary prediction equations	*R* ^2^
Equation 2	COD (mg/l) = 5974 + 4.54 TSS(mg/l) + 332 DO − 815 pH – 9 T	0.64
Equation 3	COD (mg/l) = 5785 + 4.54 TSS(mg/l) + 320 DO − 808 pH	0.64
Equation 4	BOD_5_ (mg/l) = 2300 + 2.00 TSS(mg/l) + 120 DO − 338 pH + 21 T	0.59
Equation 5	BOD_5_ (mg/l) = 2725 + 2.00 TSS(mg/l) + 148 DO − 353 pH	0.59

To investigate the significance of these variations across different sample groups and conditions, an Analysis of Variance (ANOVA) was conducted (Table 3 and S3 and 4). The ANOVA results further elucidate the influence of various factors on the measured EQVs and validate the statistical relevance of the observed differences.

**Table 3:** Analysis of variance test for regression analysis of Eq. 3.

Source	DF	SS	MS	F	*P*
Regression	3	184,976,601	61,658,867	49.33	0.000
Residual Error	97	121,242,472	1,249,922		
Total	100	306,219,073			

MSE = 1,249,922, RMSE = 1118.

**Table 4:** Analysis of variance test for regression analysis of Eq. 5.

Source	DF	SS	MS	F	*P*
Regression	3	35,774,527	11,924,842	45.61	0.000
Residual Error	97	25,360,257	261,446		
Total	100	61,134,784			

MSE = 261,446, RMSE = 511.3.

The equations presented in Table 2 indicate that Total TSS^74^ DO^16^ pH^75^ and T^76^ all influence both BOD and COD levels. However, since both BOD and COD are oxygen-related parameters, DO^16^ exhibits the highest coefficient of influence. Given that pH and DO are common independent variables in both models, a multivariate polynomial regression model may capture their simultaneous effects more accurately on both BOD and COD. Furthermore, other independent variables could potentially be excluded from the BOD/COD ratio model—known as the Biodegradability Index (BI)—since BI is primarily used as a dimensionless indicator for the relative degradability of water and wastewater quality.

### Evaluating prediction equations

**Fig. 5:**
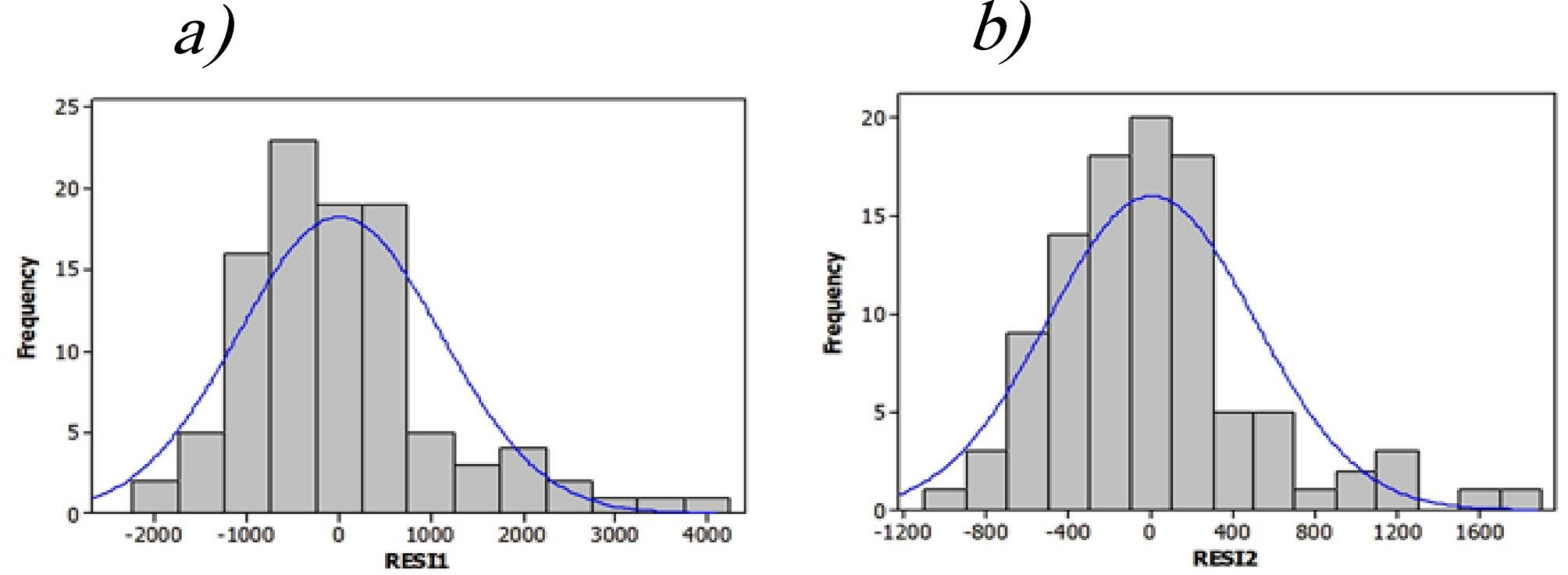
Histogram plots, displaying the frequency distribution of residuals (RES11 and RES12) for Equations of (**a**) COD and (**b**) BOD_5_.

To assess the validity of the residuals, was examined their distribution. The residuals, as shown in the Fig. 5a and b, indicate a normal distribution, supporting the assumption of normality in the regression model^77^.

Multicolinearity is a possible problem in multivariate regression that the variance inflation factor (VIF) (Kyriazos and Poga, 2023) helps one to evaluate. The VIF values presented in the output lie between 1 and 5; majority of them somewhat exceed 1 (Table S2). This suggests that the independent variables are not highly correlated, indicating that multicollinearity is unlikely to be a significant concern. (Table S2, S5). The link between BOD_5_ and COD was investigated by means of a simple linear regression analysis (Table 5).

**Table 5:** Regression analysis: BOD (mg/l) versus COD (mg/l).

regression equation	BOD (mg/l) = 222 + 0.433 COD (mg/l); *R*^2^ = 0.937			
Predictor	Coef	SE Coef	T	P
Constant	222.38	31.72	7.01	0.000
COD (mg/l)	0.43254	0.01126	38.41	0.000

**Fig. 6:**
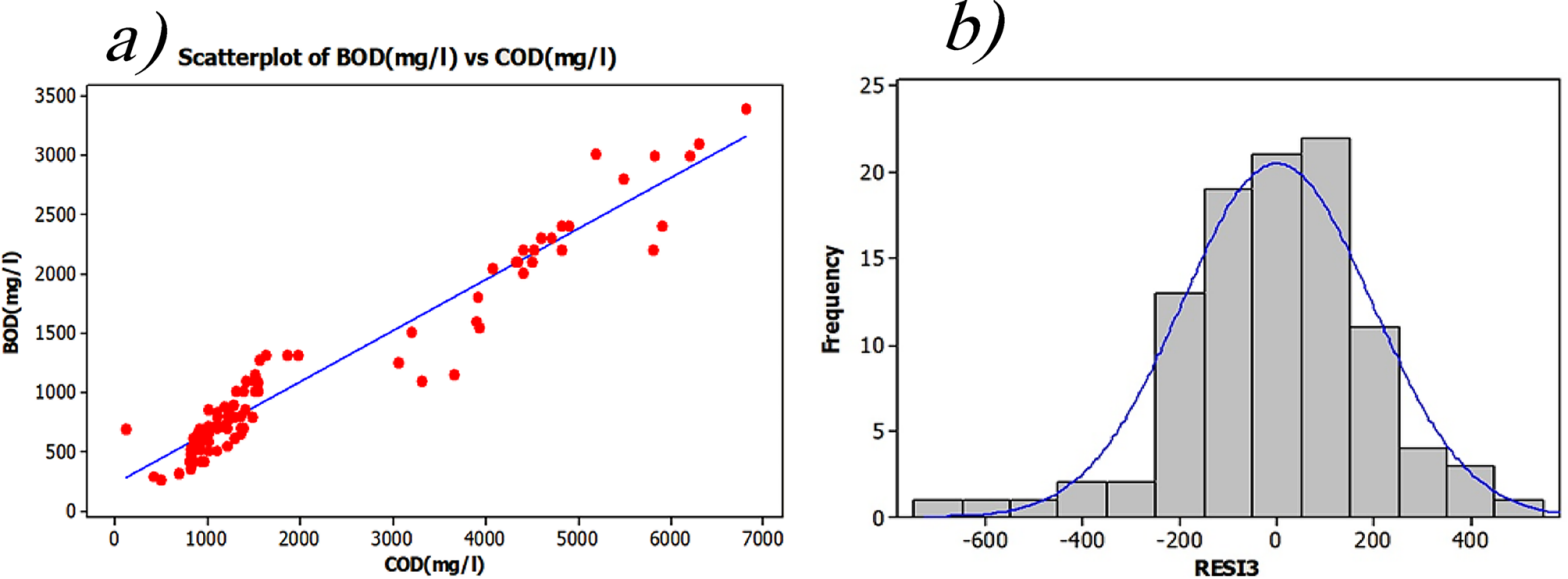
**a**) Regression between BOD_5_ and COD b) histogram plots, displaying the frequency distribution of residuals (RES11 and RES12) for Equation of BOD_5_ based on COD.

The scatter plot was initially examined visually to show the relationship between these two variables (Fig. 6a). Although this study focuses on industrial effluent, BOD₅ still contributes significantly to water quality, accounting for approximately 19% of the influence among five key water quality variables (WQVs) in certain Water Quality Indices (WQIs)^78^. While WQIs and classification systems in water resources are typically developed based on various WQVs^79^. In the context of industrial effluent, BOD₅ is often treated as an individual and significant pollution indicator. Consequently, the BI in industrial wastewater should be distinguished from that in urban or domestic wastewater, as the contributing parameters may differ. Therefore, BI in industrial contexts can be regarded as a specific component or feature within broader WQI frameworks.

This distribution chart shows the two variables’ linear relationship rather naturally. BOD_5_ is projected using simple linear regression based on COD, hence verifying their correlation (Eq. 5).

The F₀-test statistic supports the great validity of the linear regression model (Table 6); the P-value is almost zero^80^. With an R² value of 0.937, COD explains 93.7% of the fluctuation in BOD_5_.

6 $$BO{D_5}\left( {mg/l} \right) = {\text{ }}222{\text{ }} + {\text{ }}0.433{\text{ }}COD\left( {mg/l} \right){R^2} = {\text{ }}0.94$$

**Table 6:** Analysis of variance for regression between BOD (mg/l) versus COD (mg/l).

Source	DF	SS	MS	F	*P*
Regression	1	57,290,750	57,290,750	1475.48	0.000
Residual Error	99	3,844,034	38,829		
Total	100	61,134,784			

MSE = 38,829, RMSE = 197.05.

**Fig. 7:**
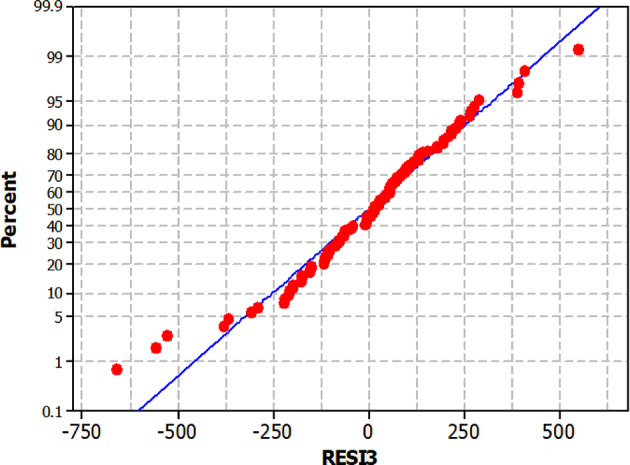
Probability plot of RESI13.

Plotting a residual histogram helps one visually see that the residuals follow a normal distribution, hence evaluating the assumption of normality (Fig. 6b). Relative BI that is appeared in this equation (Eq. 6) must not get compared with published regulatory BIs that are obtaining through theoretical oxygen demand (ThOD)^56^ Since the thresholds are variable, especially when the input data is referred to a mixed industrial wastewater. Therefore, the hypothesis regarding Industrial BI and the prediction of BOD_5_ using COD requires further profound. The Kolmogorov-Smirnov test^81^ was done to confirm the presumption of normality for the residuals and Fig. 7 shows normal probability plot. With a P-value of 0.15, the residuals show a quite normal distribution. Homoscedasticity, constant variance of residuals, is another presumption that has to be investigated to guarantee the validity of the model. The Breusch-Pagan test^82^ was run to examine this presumption. Regression of the squared residuals on the independent variable drives this test.

Homoscedasticity, referring to the constant variance of residuals, is an essential assumption that must be assessed to ensure the validity of the regression model. To evaluate this assumption, the Breusch-Pagan test was performed^82^. This test involves regressing the squared residuals on the independent variables. Homoscedasticity is confirmed if the following condition is met:

7 $$\:\frac{n}{2}{R}^{2}>{x}_{a}^{2}\left(\text{n}\right)=3.0805\to\:\frac{101}{2}\left(0.061\right)=3.0805$$

Since the critical value $\:{x}_{0.05}^{2}$(101) = 78.81, the assumption of homoscedasticity is not rejected, indicating that the variance of residuals remains constant.

### Application of the prediction equation to global contexts

**Fig. 8:**
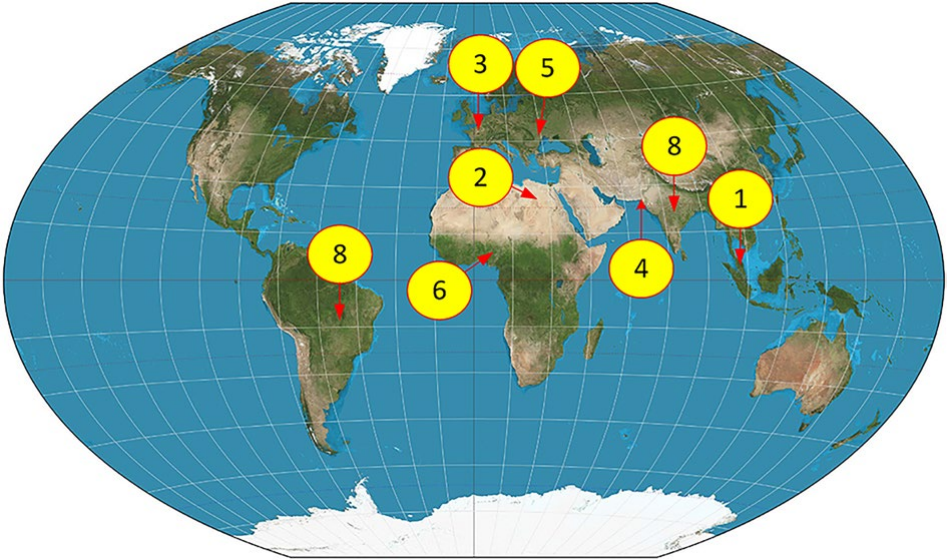
The dispersion of selected case studies for examining the application of the introduced equations. The map was generated using QGIS version 3.28 (https://qgis.org) based on OpenStreetMap data.

Finally, information from published studies carried out all around the world (Table S6) was gathered to assess the equation performance (Fig. 8). Equation 9 can efficiently predict BOD_5_ values in other countries depending on their COD values (Table 7). A prediction interval for a new observation in simple linear regression is constructed using the following Eq. 8^3^:

$$\:{\widehat{y}}_{0}-{t}_{\frac{\alpha\:}{2}}(n-2)\sqrt{MSE(1+\frac{1}{n}+\:\frac{{({x}_{0}-\stackrel{-}{x})}^{2}}{{S}_{xx}})}$$

The parameters of this equation are defined as follows:

#### $\:{x}_{0}$

The value of the independent variable for whose dependent variable we wish to forecast.

$\:{\widehat{y}}_{0}:$ predicted of $\:y$ for $\:{x}_{0}$.

#### $\:\stackrel{-}{x}$

The independent variable observations’ mean.

$\:{S}_{xx}:\:\:\:\:{S}_{xx}=\sum\:_{i=1}^{n}({x}_{i}-\stackrel{-}{x}{)}^{2}$ (The total of squared deviations from the mean for the independent observations).

#### $\:MSE={\widehat{\sigma\:}}^{2}$

mean square errors or an estimation for$\:var\left(y\right)$.

With an acceptable error margin, these equations revealed themselves to be able to forecast BOD_5_ quantities (Table 7). The best performance was seen in Kano, Nigeria^84^ where the effluent from the textile sector included chemical elements often present in TIP, so it fit the calculations. Comparably, industrial enterprises in Karachi, Sindh, Pakistan^85^ spew pollutants like oil and grease, ferric chloride (FC), and polyaluminum chloride (PAC), hence generating conditions akin to those in TIP.

**Table 7:** R^2^ value for application of prediction equation and the reliable range for case studies around the world.

No.	Case studies	Range of BOD_5_	Range of COD	Mean Interval	*R* ^2^
1	Kuala Selangor, Malaysia^86^	(405,5250)	(800,10133)	(2274,2424)	0.97
2	New Damietta and Dakahlia city- Egypt^87^	(453,1070)	(684,1526)	(710,786)	0.89
3	Pontarlier, Doubs, France^88^	(130,570)	(394,969)	(535,618)	0.81
4	Karachi, Sindh, Pakistan^85^	(524,1802)	(776,3279)	(926,1001)	0.99
5	Bucharest, Iasi and Cluj-Napoca Romania^21^	(6,750)	(18,1297)	(365,459)	0.88
6	Kano, Nigeria^84^	(665,1902)	(804,2831)	(995,1069)	0.97
7	Brazil^89^	(1092,3400)	(3054,6800)	(2173,2286)	0.88
8	India^90^	(11,255)	(80,1200)	(355,450)	0.98
	BI Average	(410.75,1874.86)			

As evidenced by the verification stage using international case study data (Table 7), the BI for industrial complexes or industrial parks consistently exceeds the threshold of 0.3. This suggests that the conventional BI models developed for individual industries may not be directly applicable to complex industrial effluents, thereby necessitating the development of a new, context-specific BI model tailored to such multifaceted wastewater streams.

## Conclusion

This study developed a robust statistical model for predicting Biological Oxygen Demand (BOD_5_) based on Chemical Oxygen Demand (COD) in industrial wastewater, specifically focusing on two major industrial parks in Tehran, Iran. Through a comprehensive one-year sampling campaign using high-frequency monitoring and standard laboratory procedures, key effluent quality variables (EQVs) such as BOD_5_, COD, DO, T, pH, and TSS were measured. The data were subjected to rigorous quality control, outlier analysis, and multivariate regression modeling, including backward elimination techniques, to derive predictive equations.

The most significant outcome of the research was the development of a simple yet highly accurate linear equation, BOD_5_ = 222 + 0.433 COD, achieving an R² of 0.94. This result confirms a strong, statistically significant relationship between COD and BOD_5_, offering a practical alternative for estimating BOD_5_ in industrial settings where direct measurement is often time-consuming and resource-intensive.

In addressing a notable gap in the literature, the study shifted focus from traditional domestic wastewater analysis to the more complex and underexplored domain of industrial effluents, which exhibit variable chemical profiles. By validating the model using wastewater datasets from eight different countries—including France, Egypt, India, Pakistan, and Nigeria—the research demonstrated the generalizability and cross-regional applicability of the approach.

The findings suggest that COD, which can be rapidly measured in the field, may serve as a reliable proxy for estimating BOD_5_, enabling more frequent and cost-effective assessments of industrial effluent quality. This is especially beneficial for industrial zones in developing countries where laboratory infrastructure may be limited.

In conclusion, this work offers a validated framework for improving wastewater monitoring efficiency through data-driven prediction models. Future research should consider extending the model to other industrial sectors and incorporating additional variables such as microbial activity or treatment system dynamics to enhance prediction accuracy. Ultimately, the model has the potential to support regulatory compliance, optimize treatment operations, and promote sustainable industrial water management practices.

## Supplementary Information

Below is the link to the electronic supplementary material.

Supplementary Material 1

## Data Availability

The datasets generated and/or analyzed during the current study are available from the corresponding authors upon reasonable request. Interested researchers may contact Professor Sadegh Partani (s_partani@ub.ac.ir) or Amin Arzhangi (a.arzhangi@stu.ub.ac.ir) to request access to the data.
