# Supplementary material for: Developing a reliable predictive model for the biodegradability index in industrial complex effluent
Source: Sci Rep. 2025 Aug 17;15:30108. doi: 10.1038/s41598-025-15866-0 (PMC12358587; doi:10.1038/s41598-025-15866-0)
Supplement: Supplementary file 1 — Supplementary Material 1 [file 41598_2025_15866_MOESM1_ESM.docx]

Backward elimination. Alpha-to-Remove: 0.1

Response is COD(mg/l) on 4 predictors, with N = 101

**Table S1** Stepwise Regression: COD(mg/l) versus TSS(mg/l), T°C, DO, PH

| 2 | 1 | step |
| --- | --- | --- |
| 5785 | 5974 | constant |
| 4.54 | 4.54 | TSS(mg/l) |
| 6.90 | 6.87 | T-Value |
| 0.000 | 0.000 | P-Value |
|  | -9 | T°C |
|  | -0.19 | T-Value |
|  | 0.849 | P-Value |
| 320 | 332 | DO |
| 2.32 | 2.16 | T-Value |
| 0.023 | 0.033 | P-Value |
| -808 | -815 | PH |
| -6.17 | -5.99 | T-Value |
| 0.000 | 0.000 | P-Value |
| 1118 | 1124 | S |
| 60.41 | 60.42 | R-Sq |

**Table S2** Regression Analysis: COD(mg/l) versus TSS(mg/l), DO, PH

| COD(mg/l) = 5785 + 4.54 TSS(mg/l) + 320 DO - 808 PH | | | | | regression equation |
| --- | --- | --- | --- | --- | --- |
| VIF | P | T | SE Coef | Coef | Predictor |
|  | 0.000 | 5.93 | 976.3 | 5785.1 | Constant |
| 1.192 | 0.000 | 6.90 | 0.6578 | 4.5418 | TSS(mg/l) |
| 1.003 | 0.023 | 2.32 | 137.9 | 319.6 | DO |
| 1.193 | 0.000 | -6.17 | 130.9 | -808.1 | PH |
| S = 1118.00 R-Sq = 60.4% R-Sq(adj) = 59.2% | | | | | |

***Table S3*** Analysis of Variance (ANOVA)

| P | F | MS | SS | DF | Source |
| --- | --- | --- | --- | --- | --- |
| 0.000 | 49.33 | 61658867 | 184976601 | 3 | Regression |
|  |  | 1249922 | 121242472 | 97 | Residual Error |
|  |  |  | 306219073 | 100 | Total |

**Table S4** Stepwise Regression: BOD(mg/l) versus TSS(mg/l), T°C, DO, PH

| 2 | 1 | step |
| --- | --- | --- |
| 2725 | 2300 | constant |
| 2 | 2 | TSS(mg/l) |
| 6.65 | 6.65 | T-Value |
| 0.000 | 0.000 | P-Value |
|  | 21 | T°C |
|  | 0.94 | T-Value |
|  | 0.349 | P-Value |
| 148 | 120 | DO |
| 2.35 | 1.72 | T-Value |
| 0.021 | 0.089 | P-Value |
| -353 | -338 | PH |
| -5.9 | -5.47 | T-Value |
| 0.000 | 0.000 | P-Value |
| 511 | 512 | S |
| 58.52 | 58.9 | R-Sq |

**Table S5** Regression Analysis: BOD(mg/l) versus TSS(mg/l), DO, PH

| BOD(mg/l) = 2725 + 2.00 TSS(mg/l) + 148 DO - 353 PH | | | | | regression equation |
| --- | --- | --- | --- | --- | --- |
| VIF | P | T | SE Coef | Coef | Predictor |
|  | 0.000 | 6.10 | 446.5 | 2724.7 | Constant |
| 1.192 | 0.000 | 6.65 | 0.3008 | 1.9998 | TSS(mg/l) |
| 1.003 | 0.021 | 2.35 | 63.07 | 148.25 | DO |
| 1.193 | 0.000 | -5.90 | 59.86 | -353.25 | PH |
|  | | | | | |

**Table S6** Prediction Interval for BOD Observations in Different Countries

| egypt-NIP | | | | | malysia-NIP | | | | |
| --- | --- | --- | --- | --- | --- | --- | --- | --- | --- |
| Prediction interval | | Predicted BOD | BOD | COD | Prediction interval | | Predicted BOD | BOD | COD |
| 830.058 | 756.870 | 793 | 720.2 | 1320.3 | 4757.05 | 4453.85 | 4605 | 5250 | 10133.33 |
| 561.830 | 475.167 | 518 | 453.3 | 684.6 | 4035.94 | 3790.83 | 3913 | 4260 | 8533.33 |
| 917.826 | 847.828 | 882 | 1070 | 1526.9 | 3315.37 | 3127.27 | 3221 | 3450 | 6933.33 |
| 911.485 | 841.280 | 876 | 937 | 1512 | 2835.61 | 2684.30 | 2759 | 2250 | 5866.67 |
|  |  |  |  |  | 1880.40 | 1793.99 | 1837 | 1500 | 3733.33 |
|  |  |  |  |  | 948.97 | 879.92 | 914 | 960 | 1600 |
|  |  |  |  |  | 610.35 | 526.47 | 658 | 405 | 800 |
|  |  |  |  |  | 1009.55 | 942.09 | 975 | 529 | 1741.9 |

| pakistan-NIP | | | | | Franc-NIP | | | | |
| --- | --- | --- | --- | --- | --- | --- | --- | --- | --- |
| Prediction interval | | Predicted BOD | BOD | COD | Prediction interval | | Predicted BOD | BOD | COD |
| 1678.84 | 1602.52 | 1640 | 1802 | 3279 | 638.976 | 556.677 | 597 | 411 | 868 |
| 1165.50 | 1100.26 | 1132 | 1236 | 2105 | 681.535 | 601.491 | 641 | 496 | 969 |
| 1580.39 | 1508.06 | 1544 | 1598 | 3056 | 654.560 | 573.101 | 613 | 460 | 905 |
| 665.52 | 584.64 | 625 | 607 | 931 | 439.897 | 345.708 | 392 | 130 | 394 |
| 969.01 | 900.54 | 934 | 953 | 1647 | 600.677 | 516.254 | 558 | 570 | 777 |
| 665.94 | 585.08 | 625 | 524 | 932 | 625.503 | 542.467 | 583 | 530 | 836 |
| 1227.29 | 1162.17 | 1194 | 1272 | 2248 | 677.741 | 597.500 | 637 | 460 | 960 |
| 737.25 | 659.97 | 698 | 748 | 1101 | 598.574 | 514.032 | 556 | 340 | 772 |
|  |  |  |  |  | 573.346 | 487.355 | 530 | 306 | 712 |
|  |  |  |  |  | 683.644 | 603.708 | 643 | 440 | 974 |
|  |  |  |  |  | 628.029 | 545.132 | 586 | 462 | 842 |
|  |  |  |  |  | 624.240 | 541.135 | 582 | 452 | 833 |

| India -NIP | | | | |
| --- | --- | --- | --- | --- |
| Prediction interval | | Predicted BOD | BOD | COD |
| 779.104 | 703.755 | 741 | 255.1 | 1,200 |
| 673.525 | 593.065 | 632 | 210.5 | 950 |
| 308.482 | 205.488 | 256 | 11.3 | 80 |
| 313.917 | 211.299 | 262 | 96 | 93 |
| 372.059 | 273.403 | 322 | 21 | 232 |
| 378.337 | 280.102 | 329 | 13 | 247 |
| 321.861 | 219.792 | 270 | 42 | 112 |

**Table S7** PIP Dataset

| PIP | | | | | |
| --- | --- | --- | --- | --- | --- |
| BOD(mg/l) | COD(mg/l) | TSS(mg/l) | DO | T°C | PH |
| 875.00 | 1180 | 120 | 22 | 2.3 | 6.4 |
| 1000 | 1540 | 130 | 24 | 1.5 | 6.3 |
| 1100 | 1530 | 230 | 21.6 | 1.1 | 6.7 |
| 1310 | 1610 | 250 | 21.5 | 1.1 | 6.5 |
| 1280 | 1550 | 280 | 19 | 1.5 | 7 |
| 530 | 875 | 250 | 15 | 2 | 7.2 |
| 256 | 485 | 120 | 13 | 2.5 | 7.35 |
| 310 | 679 | 195 | 18 | 2.9 | 6.9 |
| 524 | 850 | 210 | 18.5 | 2 | 6.8 |
| 578 | 1000 | 285 | 19 | 2.3 | 7 |
| 857 | 1230 | 300 | 20 | 2.3 | 6.9 |
| 670 | 1000 | 485 | 21 | 2.5 | 8.22 |
| 1310 | 1850 | 300 | 19.5 | 2.5 | 8 |
| 1100 | 1500 | 250 | 18 | 2.7 | 7.8 |
| 690 | 1100 | 180 | 19 | 2.7 | 7.5 |
| 600 | 980 | 200 | 19.5 | 2.5 | 6.8 |
| 710 | 1100 | 280 | 19.5 | 2.8 | 7.04 |
| 510 | 850 | 300 | 19 | 2.7 | 7.25 |
| 600 | 838 | 200 | 18 | 2.7 | 7.32 |
| 680 | 895 | 195 | 19.5 | 2.4 | 7.17 |
| 510 | 873 | 180 | 19.5 | 2.2 | 7.1 |
| 630 | 875 | 200 | 20 | 2.1 | 7 |
| 710 | 1000 | 250 | 20.5 | 2 | 7.24 |
| 700 | 981 | 180 | 20 | 2 | 6.7 |
| 510 | 910 | 30 | 18.5 | 1.4 | 7.2 |
| 550 | 900 | 45 | 18 | 1.7 | 6.9 |
| 780 | 1100 | 180 | 17.5 | 1.5 | 6.95 |
| 710 | 1000 | 100 | 18 | 2 | 7 |
| 650 | 890 | 85 | 18.2 | 2.5 | 7 |
| 800 | 1350 | 40 | 17.7 | 2.5 | 7.27 |
| 1150 | 1510 | 55 | 18.5 | 2.2 | 6.3 |
| 1000 | 1300 | 80 | 17.5 | 2.8 | 6.2 |
| 1000 | 1380 | 90 | 18 | 2.5 | 7 |
| 780 | 1480 | 95 | 18.5 | 2 | 6.91 |
| 700 | 1380 | 140 | 17.5 | 2.5 | 7.2 |
| 600 | 1280 | 120 | 16.5 | 1.5 | 7.35 |
| 650 | 1000 | 150 | 16 | 1 | 7.5 |
| 281 | 409 | 60 | 15.5 | 0.6 | 6.8 |
| 530 | 830 | 85 | 15.5 | 0.7 | 7.36 |
| 410 | 785 | 60 | 15.5 | 0.5 | 7.1 |
| 500 | 1000 | 180 | 16.5 | 0.5 | 7 |
| 680 | 110 | 280 | 16.5 | 0.5 | 6.9 |
| 1000 | 1500 | 350 | 16 | 0.6 | 6.8 |
| 1310 | 1960 | 420 | 15.8 | 0.8 | 6.8 |
| 780 | 1280 | 385 | 17.2 | 1.3 | 7.35 |
| 1100 | 1410 | 420 | 16.5 | 0.5 | 6.94 |
| 700 | 1350 | 150 | 17.5 | 0 | 6.2 |
| 750 | 1200 | 400 | 17 | 0 | 6.1 |
| 850 | 1000 | 400 | 16.5 | 0.7 | 7.12 |
| 890 | 1266 | 680 | 17 | 1 | 7.12 |
| 500 | 1100 | 500 | 16.7 | 0.7 | 7 |
| 650 | 1350 | 380 | 15.5 | 0.5 | 7 |
| 1080 | 1536 | 280 | 15.2 | 0.3 | 6.37 |
| 1000 | 1500 | 250 | 15.5 | 0.3 | 6.5 |
| 1000 | 1380 | 200 | 16 | 0.5 | 7.2 |
| 850 | 1395 | 300 | 16.5 | 0.3 | 6.5 |
| 800 | 1250 | 150 | 16 | 0.1 | 6.74 |
| 800 | 1200 | 180 | 15.2 | 0.1 | 6.8 |
| 830 | 1100 | 100 | 14.7 | 0.1 | 6.8 |
| 543 | 1200 | 110 | 14.2 | 0.5 | 7 |
| 710 | 1150 | 180 | 15 | 0.3 | 6.5 |
| 410 | 950 | 180 | 15.5 | 0.5 | 6.97 |
| 510 | 810 | 100 | 15.7 | 0.3 | 7 |
| 480 | 800 | 100 | 16.5 | 0.1 | 6.8 |
| 400 | 830 | 200 | 17.2 | 0.1 | 7.2 |
| 650 | 1000 | 380 | 16.8 | 0.5 | 6.87 |
| 430 | 830 | 350 | 15 | 0.3 | 6.7 |
| 350 | 800 | 65 | 10.5 | 0.5 | 7.21 |
| 410 | 920 | 200 | 16 | 0.3 | 6.42 |
| 700 | 1200 | 150 | 16 | 0.2 | 7.41 |
| 610 | 1000 | 180 | 16 | 0.1 | 7.25 |

**Table S8** NIP Dataset

| NIP | | | | | |
| --- | --- | --- | --- | --- | --- |
| BOD(mg/l) | COD(mg/l) | TSS(mg/l) | T°C | DO | PH |
| 3100 | 6300 | 232 | 0 | 24 | 6 |
| 2200 | 4400 | 320 | 0 | 23 | 8.5 |
| 2400 | 4810 | 350 | 0 | 24 | 5.21 |
| 2100 | 4320 | 391 | 0 | 24 | 4.8 |
| 3000 | 5810 | 360 | 0 | 23 | 5.5 |
| 2100 | 4500 | 1191 | 0 | 20 | 6.23 |
| 1800 | 3910 | 490 | 0 | 22 | 4.21 |
| 2050 | 4310 | 430 | 0 | 23 | 7.12 |
| 3010 | 5180 | 500 | 0 | 23 | 3.12 |
| 2800 | 5480 | 610 | 0 | 22 | 5.21 |
| 2400 | 5890 | 690 | 0 | 19 | 4.05 |
| 2200 | 4812 | 510 | 0 | 18 | 6.75 |
| 2010 | 4400 | 432 | 0 | 17 | 5.12 |
| 1500 | 3200 | 366 | 0 | 15 | 8.19 |
| 2200 | 4510 | 420 | 0 | 16 | 6.12 |
|  | 5800 | 505 | 0 | 17 | 5.7 |
| 3000 | 6200 | 500 | 0 | 17 | 5.32 |
| 2400 | 4900 | 380 | 0 | 16 | 4.61 |
| 1600 | 3900 | 310 | 0 | 17 | 5.27 |
| 2200 | 4510 | 490 | 0 | 16 | 5.3 |
| 1250 | 3054 | 258 | 0 | 17 | 6.3 |
| 2300 | 4700 | 336 | 0 | 16 | 7.3 |
| 2200 | 4400 | 320 | 0 | 11 | 7.2 |
| 2400 | 4890 | 638 | 0 | 14 | 6.12 |
| 3400 | 6800 | 680 | 0 | 17 | 4.63 |
| 2100 | 4330 | 492 | 0 | 15 | 5.12 |
| 2400 | 4810 | 530 | 0 | 15 | 5.81 |
| 1540 | 3930 | 412 | 0 | 16 | 4.6 |
| 2300 | 4600 | 540 | 0 | 16 | 7.56 |
| 2040 | 4074 | 558 | 0 | 17 | 7.65 |
|  | 5320 | 660 | 0 | 15 | 6.98 |
| 1092 | 3300 | 330 | 0 | 17 | 7.7 |
| 1140 | 3650 | 310 | 0 | 18 | 6.03 |
